# PI3KC2β depletion rescues endosomal trafficking defects in *Mtm1* knockout skeletal muscle cells

**DOI:** 10.1016/j.jlr.2025.100756

**Published:** 2025-02-12

**Authors:** Mélanie Mansat, Afi Oportune Kpotor, Anne Mazars, Gaëtan Chicanne, Bernard Payrastre, Julien Viaud

**Affiliations:** 1 INSERM UMR1297, University of Toulouse 3, Institute of Metabolic and Cardiovascular Diseases (I2MC), Toulouse, France; 2 University Hospital of Toulouse, Hematology Laboratory, Toulouse, France

**Keywords:** phosphoinositide, phospholipids/trafficking, cell signaling, muscle

## Abstract

Phosphoinositides constitute a class of seven phospholipids found in cell membranes, regulating various cellular processes like trafficking and signaling. Mutations in their metabolizing enzymes are implicated in several pathologies, including X-linked myotubular myopathy, a severe myopathy caused by mutations in the *MTM1* gene. MTM1 (myotubularin 1) acts as a phosphoinositide 3-phosphatase, targeting PI3P (phosphatidylinositol 3-phosphate) and phosphatidylinositol 3,5-bisphosphate, crucial for endolysosomal trafficking. Studies in X-linked myotubular myopathy animal models have demonstrated that loss of MTM1 results in PI3P accumulation in muscle. Moreover, inactivating the class II phosphoinositide 3-kinase beta rescues the pathological phenotype and decreases PI3P levels, suggesting that the normalization of PI3P levels could be responsible for that rescue mechanism. In this study, using an *Mtm1*-KO skeletal muscle cell line, we investigated the localization of the PI3P pool metabolized by MTM1 in endosomal compartments. Our findings reveal that MTM1 metabolizes a pool of PI3P on EEA1 (early endosome antigen 1)-positive endosomes, leading to impaired Rab4 recycling vesicle biogenesis in the absence of MTM1. Furthermore, depletion of class II phosphoinositide 3-kinase beta rescued *Mtm1*-KO cell phenotype, normalized PI3P level on EEA1-positive endosomes, and restored Rab4-positive vesicle biogenesis. These results indicate that MTM1 is critical for the homeostasis of endosomal trafficking, and that depletion of MTM1 potentially alters cargo recycling through Rab4-positive vesicle trafficking.

## Graphical Abstract

**Figure:**
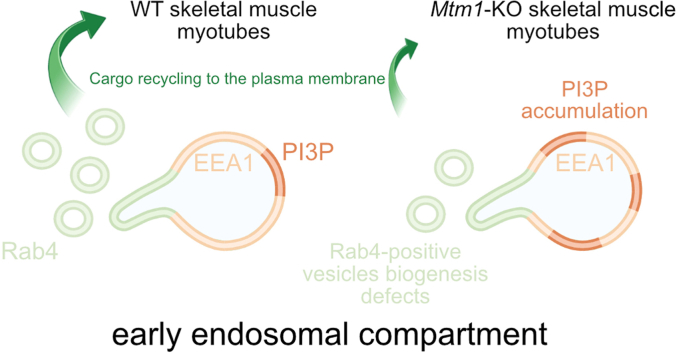

Phosphoinositides are a class of phospholipids present in cellular membranes, characterized by an inositol head group attached to a glycerol-based lipid backbone (Fig. 1A). Their structure consists of various phosphorylation states at three different positions on the inositol ring, governed by the action of specific phosphoinositide kinases and phosphatases, yielding to seven phosphoinositide species: phosphatidylinositol 3-phosphate (PI3P), phosphatidylinositol 4-phosphate, phosphatidylinositol 5-phosphate, phosphatidylinositol 3,4-bisphosphate, phosphatidylinositol 3,5-bisphosphate (PI(3,5)P_2_), phosphatidylinositol 4,5-bisphosphate, and phosphatidylinositol 3,4,5-trisphosphate (1) (Fig. 1A). These molecules serve as key signaling hubs within the cell membranes, regulating a plethora of cellular processes. Their functions encompass mediating intracellular trafficking, modulating cytoskeletal dynamics, regulating ion channel activity, and orchestrating cell proliferation and survival pathways (2). Phosphoinositides achieve this by recruiting effector proteins to specific cellular membranes, thereby influencing membrane curvature, protein localization, and signaling events. The dynamic interplay between the synthesis, degradation, and spatial distribution of phosphoinositides underpins the complexity and versatility of their cellular functions, making them indispensable players in cellular signaling and membrane biology. Particularly, PI3P and PI(3,5)P_2_ are critical regulators of endosomal trafficking, a process crucial for maintaining cellular homeostasis and regulating signaling pathways (3, 4). Importantly, they dictate the identity and behavior of endosomal compartments, influencing sorting, fusion, and trafficking events. PI3P is generated primarily by the class II and III phosphatidylinositol 3-kinases (PI3Ks) from phosphatidylinositol (PI) (Fig. 1B), serving as a key determinant in the early endosomal pathway. It facilitates the recruitment of effector proteins involved in cargo selection and membrane deformation, promoting endosomal maturation and fusion events. Conversely, PI(3,5)P_2,_ synthesized by the PI3P 5-kinase PIKfyve (Fig. 1B), plays a pivotal role in late endosomal trafficking and lysosome biogenesis. It regulates the formation of tubular structures on late endosomes, facilitating cargo transport and promoting fusion with lysosomes (5). Overall, the dynamic interplay between PI3P and PI(3,5)P_2_ orchestrates the sequential progression of endosomal maturation, ensuring efficient cargo sorting, vesicular trafficking, and organelle biogenesis within the endolysosomal system (6).

**Fig. 1:**
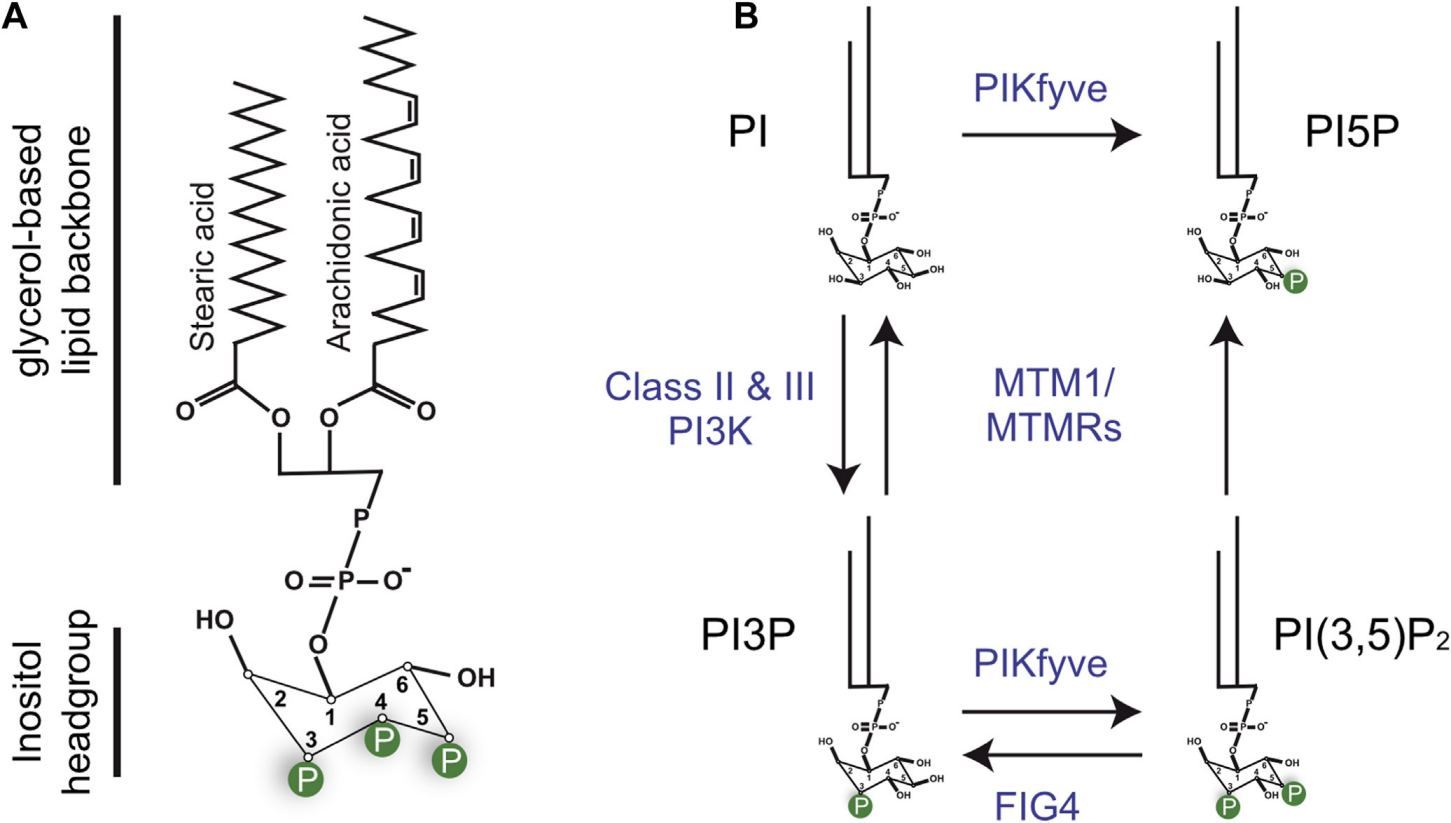
Phosphoinositides, structure and metabolism. A: Structure of phosphoinositides. The positions 3, 4, and 5 in the inositol headgroup can be phosphorylated (labeled with a green “P”) by specific kinases, and dephosphorylated by specific phosphatases, to produce seven different phosphoinositides. B: Phosphoinositide metabolism in which MTM1 is implicated.

The pivotal roles of phosphoinositides in cell functions are highlighted by the direct implication of the phosphoinositide-metabolizing enzymes in various pathologies. For instance, mutations in phosphoinositide kinases and phosphatases have been linked to cancer, neurodegenerative disorders, and immune dysfunctions (1). Therefore, understanding the intricate regulation of phosphoinositide-metabolizing enzymes and their roles in disease pathology holds promise for the development of targeted therapeutic strategies aimed at restoring normal phosphoinositide signaling and mitigating disease progression. An example of such disease is X-linked myotubular myopathy (XLMTM), a severe muscle disorder characterized by muscle weakness and atrophy, which is due to mutations in the *MTM1* gene (7). This gene encodes for the phosphoinositide 3-phosphatase myotubularin 1 (MTM1), which plays a crucial role in regulating membrane dynamics and vesicle trafficking within cells (8). It specifically targets PI3P and PI(3,5)P_2_ (9, 10) (Fig. 1B), two key phosphoinositides of the endolysosomal trafficking (2). Accordingly, the loss of MTM1 leads to an accumulation of PI3P in the muscle (11, 12, 13). Studies on *Drosophila melanogaster*, in which *mtm*, the sole ortholog of human MTM1/MTMR2, was knocked out, showed that the depletion of the sole class II (*Pi3K68D*), but not class III PI3K (*Vps34*), rescued myotubularin-dependent muscle phenotypes (14) (Fig. 1B). Class II PI3K specifically generates PI3P from PI and phosphatidylinositol 3,4-bisphosphate from phosphatidylinositol 4-phosphate (15), suggesting that *Pi3K68D* depletion counteract PI3P accumulation observed in *mtm* null flies to rescue the observed phenotypes. More recent studies on mouse models have shown that loss of expression of the class II phosphoinositide 3-kinase β (*Pik3c2b*) (16), or inactivation of its lipid kinase activity, ameliorated the phenotypes of an XLMTM mouse model (12, 17). Importantly, class III PI3K (*Pik3c3*) deletion exacerbates the *Mtm1*-KO phenotype (17), suggesting that MTM1 and PI3KC2β regulate a specific and common pool of PI3P. While the question is central to better understand the molecular basis of this disease, where this pool is localized in mammalian muscle cells is unknown.

Recently, we developed an *Mtm1*-KO skeletal muscle cell line that recapitulates XLMTM features (18, 19). In the current study, we took advantage of this cell line to determine whether there is a shared pool of PI3P metabolized by MTM1 and PI3KC2β within endosomal compartments and to assess any modifications in these compartments. As expected, depletion of PI3KC2β rescues the phenotype of *Mtm1*-KO skeletal muscle cells. We found that in the absence of MTM1, PI3P accumulates on early endosome antigen 1 (EEA1)-positive vesicles, with no changes in their number, and that depletion of PI3KC2β restored this PI3P pool. Surprisingly, our results indicate that PI3P is primarily localized on Rab4-positive vesicles in C2C12 myotubes, and the absence of MTM1 results in impaired Rab4 vesicle biogenesis, a phenotype that is also rescued by depletion of PI3KC2β. These findings suggest that MTM1 plays a critical role in controlling a specific pool of PI3P that maintains the homeostasis of endosomal compartments, potentially impacting cargo recycling.

## Materials and Methods

### Cell culture

WT and *Mtm1*-KO C2C12 myoblasts (18) were cultured in DMEM-glutamax without pyruvate (Gibco) supplemented with 20% fetal bovine serum. When cells reached 90%–95% confluency, medium was changed to DMEM-glutamax without pyruvate supplemented with 2% horse serum to start cell differentiation. Cell differentiation was done on glass coverslips coated with 0.2% gelatin. HeLa cells (ATCC) were cultured in DMEM-glutamax (Gibco) supplemented with 10% fetal bovine serum. HeLa cells were transfected with jetPEI (Polyplus) according to the manufacturer's protocol.

Cells were routinely tested for *Mycoplasma* contamination, and all tests were negative.

### Antibodies and reagents

All reagents were purchased from commercial sources; Accutase (BD Biosciences), DMEM glutaMAX (without pyruvate) (Gibco), Dulbecco's PBS (Eurobio Scientific), fetal bovine serum (Gibco), cell lysis buffer (10X) (Cell Signaling), gelatin from porcine skin (type A), formaldehyde, glutaraldehyde, digitonin, Triton X-100, goat serum, NH_4_Cl, Pipes (all from Sigma-Aldrich), FluorSave (Calbiochem), and 4',6-diamidino-2-phenylindole (Euromedex). Antibodies against the following proteins were used in this study: Rab4 (ThermoFisher, Research Resource Identifier [RRID]: AB_2269382), Rab5 (Cell Signaling; RRID: AB_2300649), EEA1 (ENZO, ALX-210-239-C100), PI3KC2β (BD Transduction Laboratories; RRID: AB_398865), Myosin Heavy Chain (MYH4) (eBioscience; RRID: AB_2572894), HA (Sigma-Aldrich; catalog no.: H3663; RRID: AB_262051), FLAG (Sigma-Aldrich; catalog no.: F3165; RRID: AB_259529), anti-rabbit Alexa 488 (Thermo Fisher Scientific), and antimouse Alexa 488 (Thermo Fisher Scientific). shRNA constructs include mouse *Pik3c2b* (stock: TRCN0000360889 [*Pik3c2b* #1], stock: TRCN0000360890 [*Pik3c2b* #2]). TRC shRNAs were from Sigma-Aldrich (St. Louis, MO); SHC002 (Sigma-Aldrich) was used as a control.

### Cloning, mutagenesis, and recombinant protein purification

The plasmid coding for GST-mCherry-FYVE-domain (Hrs) was described previously (20). GST-mCherry-FYVE-domain (Hrs mut) that does not bind PI3P (R24A/K25A/R29A) was obtained by directed mutagenesis using the following primers: Forward: CGGGGTGATGACCGCTGCGGCCCACTGCGCGGCGTGTGGGCAG, Reverse: CTGCCCACACGCCGCGCAGTGGGCCGCAGCGGTCATCACCCCG. Both proteins were expressed in BL21(DE3) bacteria overnight at 18°C using 0.5 mM IPTG and purified by affinity chromatography using Glutathione Sepharose 4B beads (GE Healthcare) according to the manufacturer's instructions. Both proteins were purified in 50 mM Tris at pH 8.0, 100 mM NaCl, 10% glycerol, snap-frozen, and stored at −80°C. N174-MCS (puromycin) was a gift from Adam Karpf (Addgene plasmid #81068; http://n2t.net/addgene:81,068; RRID: Addgene_81068). pcDNA3 HA-PI3KC2β was kindly provided by V. Haucke (Leibniz Forschungsinstitut für Molekulare Pharmakologie [FMP], Berlin, Germany). The N174(puro)-HA-PI3KC2β construct was generated using the In-Fusion cloning kit (Takara Bio) following the manufacturer's protocol. The HA-PI3KC2β insert was amplified using the following primers: Forward: 5′-CGTGAGGATCGAATTCATGGCGTACCCATACGACGT-3′ and Reverse: 5′-CGGTAGAATTGGATCCTTACAAGGTGCCATGACTTCGA-3'. The N174-MCS (puromycin) vector was linearized using BamHI and EcoRI. The plasmid N174(puro)-FLAG-MTM1 was described previously (18).

### Lentivirus production and transduction

Lentiviruses were produced as previously described (21). C2C12 cells were transduced by incubation with lentiviral particles and then washed 12 h later. After 72 h, cells were selected in the presence of 2 μg ml^−1^ puromycin for 4 days.

### Western blotting

Total cellular proteins were extracted with Cell Lysis Buffer (Cell Signaling), and 20 μg proteins were separated by electrophoresis on 4%–12% gradient SDS-polyacrylamide gel (Life Technologies) and transferred on Immobilon-P membranes (Millipore). Membranes were then incubated with appropriate antibodies, and immunoreactive bands were detected by chemiluminescence using ChemiDoc MP (Bio-Rad Laboratories) with clarity Western ECL substrate detection system (Bio-Rad Laboratories). Full-length original Western blots are provided in supplemental data section.

### Microscopy and image analysis

Cells were fixed with 3.7% formaldehyde, quenched with NH_4_Cl for 10 min, and permeabilized with 20 μM digitonin in Pipes-BS (Pipes 20 mM, pH 6.8, NaCl 137 mM, and KCl 2.7 mM) for 5 min. After a 1 h saturation period in 10% goat serum/Pipes-buffered saline, cells were incubated with 50 μg/ml of the GST-mCherry-FYVE-domain (Hrs) probe and primary antibodies for 2 h at room temperature. After three washes with Pipes-BS, secondary fluorescent antibodies in 10% goat serum/Pipes-BS were added for 1 h, washed three times with Pipes-BS. Cells were then fixed a second time with 3.7% formaldehyde, nuclei were stained with diamidino-2-phenylindole, washed three times with Dulbecco's PBS, mounted with FluorSave reagent. Imaging was performed with confocal LSM780 Zeiss microscope (Zen software; x63 objective). Images were analyzed using Fiji. Protein signal colocalization (using both Pearson's and Mander's coefficients) was computed with the JaCoP Fiji plugin. Quantification of vesicle density was done with a custom Fiji plugin.

### Statistics and reproducibility

Statistical analyses were performed as described for each experiment in the figure legends using GraphPad Prism 9 (GraphPad Software, Inc). All data were represented as the mean ± 95% CI. In all tests and all statistical analyzed datasets, the levels of significance were defined as: ∗*P* < 0.05, ∗∗*P* < 0.01, ∗∗∗*P* < 0.001, and ∗∗∗∗*P* < 0.0001. For comparisons between two experimental groups, data were analyzed by Student's *t*-test. For comparisons involving more than two experimental groups, data were analyzed using one-way ANOVA followed by Šídák's multiple comparisons test. Each experiment was conducted with at least three independent replications, yielding similar results.

## Results

### Knockdown of *Pik3c2b* rescues the phenotype of *Mtm1*-KO myotubes and reduces PI3P accumulation

**Fig. 2:**
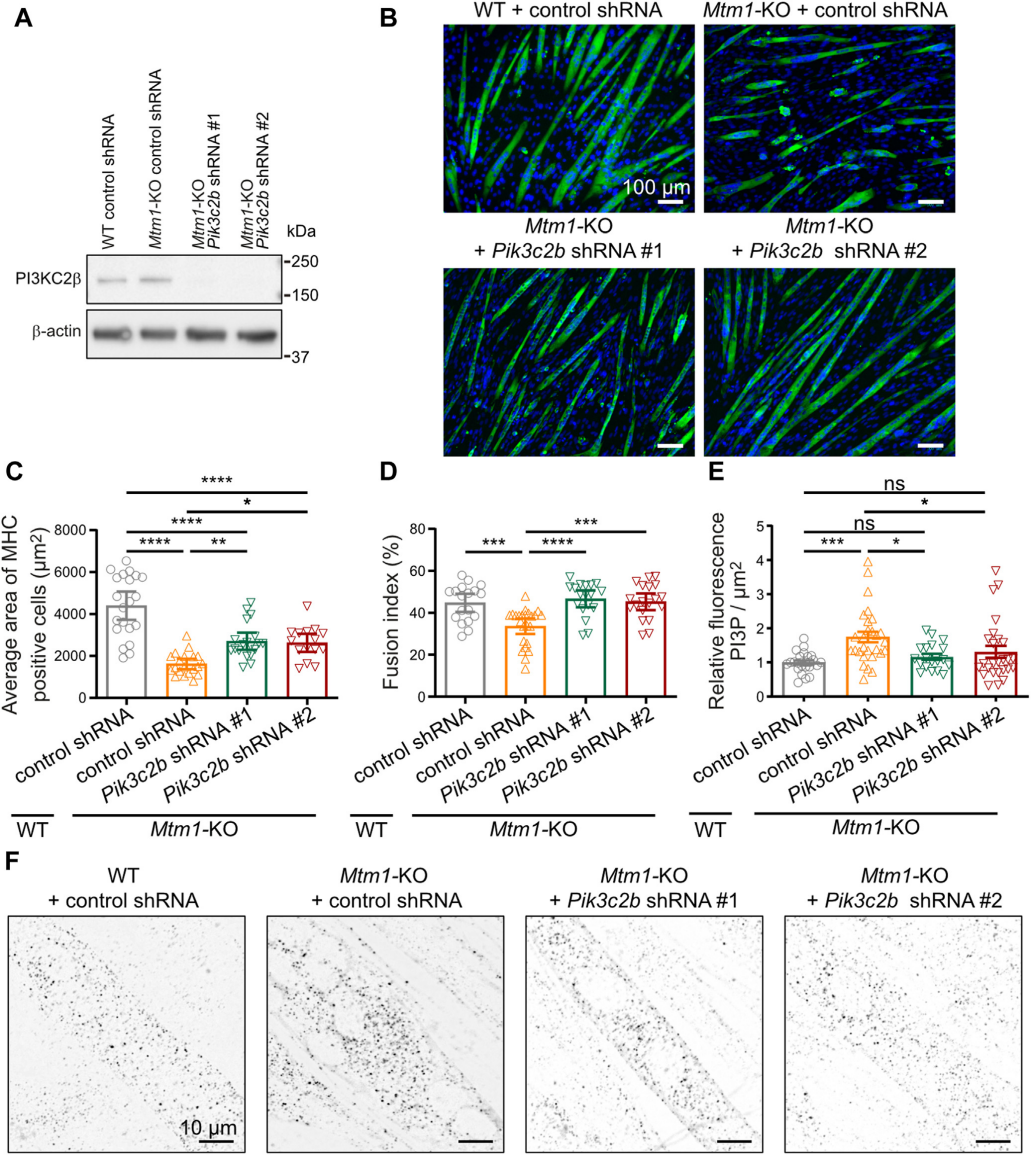
Knocking down *Pik3c2b* rescues *Mtm1*-KO phenotypes. A: Expression of PI3KC2β and actin in WT and *Mtm1*-KO C2C12 myotubes expressing control shRNA, *Pik3c2b* shRNA #1, *Pik3c2b* shRNA #2 analyzed by Western blot. Images are representative of three independent experiments. B: MYH4 (green) and DAPI (blue) staining of C2C12 WT and *Mtm1*-KO expressing control shRNA and *Mtm1*-KO expressing *Pik3c2b* shRNA #1, *Pik3c2b* shRNA #2 at 6 days of differentiation (scale bar represents 100 μm). C: Quantification of average area of MYH4 labeled WT and *Mtm1*-KO myotubes from the experiment described in B. Data are represented as mean ± 95% CI, n = 4, five fields per independent experiment, each point represents one field of view, ∗*P* < 0.05, ∗∗*P* < 0.01, and ∗∗∗∗*P* < 0.0001 according to one-way ANOVA test and Šídák's multiple comparisons test. D: Quantification of the fusion index for the WT and *Mtm1*-KO cells from the experiment described in B. Data are represented as mean ± 95% CI, n = 4, five fields per independent experiment, each point represents one field of view, ∗∗∗*P* < 0.001, ∗∗∗∗*P* < 0.0001 according to one-way ANOVA test and Šídák's multiple comparisons test. E: Quantification of PI3P in WT and *Mtm1*-KO C2C12 myotubes expressing control shRNA, and *Mtm1*-KO expressing *Pik3c2b* shRNA #1, *Pik3c2b* shRNA #2 using the FYVE domain of Hrs protein as a probe for PI3P. Data are represented as mean ± 95% CI, n = 4, five fields per independent experiment, each point represents one field of view, ns = not significant, ∗∗∗*P* < 0.001, according to one-way ANOVA test and Šídák's multiple comparisons test. F: Representative images of PI3P labeling of WT and *Mtm1*-KO C2C12 myotubes expressing control shRNA, and *Mtm1*-KO expressing *Pik3c2b* shRNA #1, *Pik3c2b* shRNA #2 at day 6 of differentiation. Scale bar represents 10 μm.

In order to determine if *Pik3c2b* knockdown can rescue *Mtm1*-KO myotube phenotypes, as shown in animal models (12, 17), we used lentiviruses expressing shRNA. Efficient knockdown was achieved with *Pik3c2b* shRNA #1 and #2 (Fig. 2A and supplemental Fig. S1), leading to a rescue of *Mtm1*-KO myotube phenotypes (Fig. 2B). The average area of myotubes was decreased in *Mtm1*-KO myotubes, as previously described (18), and both *Pik3c2b* shRNAs resulted in an increase of myotube area (Fig. 2C). The fusion index (percentage of nuclei in MYH4-positive cells containing more than two nuclei) was also decreased in *Mtm1*-KO myotubes expressing a control shRNA, with a concomitant rescue observed with both *Pik3c2b* shRNAs (Fig. 2D). We next quantified PI3P levels by immunofluorescence. To localize PI3P, we used the well-characterized FYVE domain of the Hrs protein fused to mCherry, purified it as a recombinant protein to circumvent potential pitfalls linked to the utilization of transfected phosphoinositide probes (21, 22, 23) (supplemental Fig. S2). As already described, PI3P levels elevated in *Mtm1*-KO myotubes (18), similar to what has been observed in muscle from *Mtm1*-KO animal models (11, 13), and both *Pik3c2b* shRNAs resulted in a rescue in PI3P levels (Fig. 2E, F). These results confirm that the C2C12 *Mtm1*-KO cell line recapitulates what has been observed in XLMTM animal models (18), including the observation that knocking down *Pik3c2b* rescues the phenotype of *Mtm1* KO, potentially by reducing the accumulation of PI3P.

### Localization of the PI3P pool hydrolyzed by MTM1 in myotubes

**Fig. 3:**
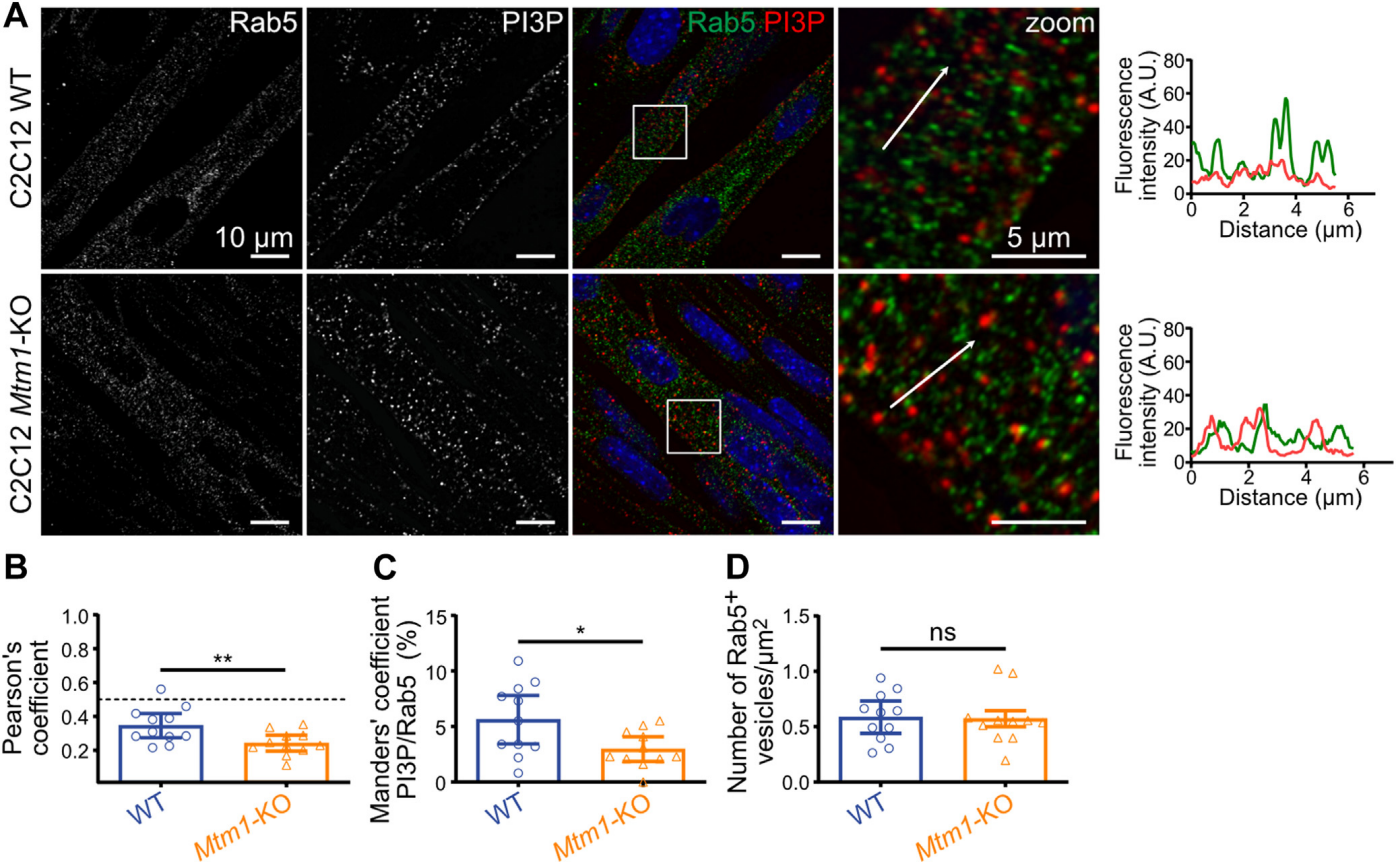
The absence of MTM1 depletes PI3P from Rab5-positive vesicles in myotubes. A: Representative confocal images of C2C12 WT and *Mtm1*-KO cells at 6 days of differentiation, labeled with a probe for PI3P (FYVE domain of Hrs protein), Rab5, and DAPI (blue). Scale bar represents 10 μm. Magnification of the boxed area shown on the right. Scale bar represents 5 μm. Respective line scans are shown on the right of the zoom panel. B: Quantification of Pearson's coefficient from the experiment described in A. Results are shown as mean ± 95% CI, n = 3, each point represents a field. ∗∗*P* < 0.01 according to Student's *t*-test. C: Quantification of Manders' coefficient from the experiment described in A. Results are shown as mean ± 95% CI, n = 3, each point represents one field of view. ∗*P* < 0.05 according to Student's *t*-test. D: Quantification of the number of Rab5-positive vesicles from the experiment described in A. Results are shown as mean ± 95% CI, n = 3, each point represents one field of view. ns = not significant according to Student's *t*-test.

PI3P and active Rab5 work closely together to recruit various effector proteins to the membranes of early endosomes through a mechanism known as coincidence detection (24, 25). Therefore, we first assessed PI3P localization on Rab5-positive vesicles on WT and *Mtm1*-KO myotubes at 6 days of differentiation. As expected, both labeled intracellular vesicular structures distinct from the nuclei (Fig. 3A). However, analysis using Pearson's coefficient indicated a weak correlation between the two molecules, suggesting that Rab5-positive vesicles are not the primary site of PI3P localization in this cell system (Fig. 3B). No increase in PI3P on Rab5-positive vesicles was observed in *Mtm1*-KO myotubes, suggesting that MTM1 does not hydrolyze PI3P into PI on these vesicles (Fig. 3C). Furthermore, quantifications of Rab5-positive vesicle density indicated no significant alteration in this endocytic compartment in the absence of MTM1 (Fig. 3D).

**Fig. 4:**
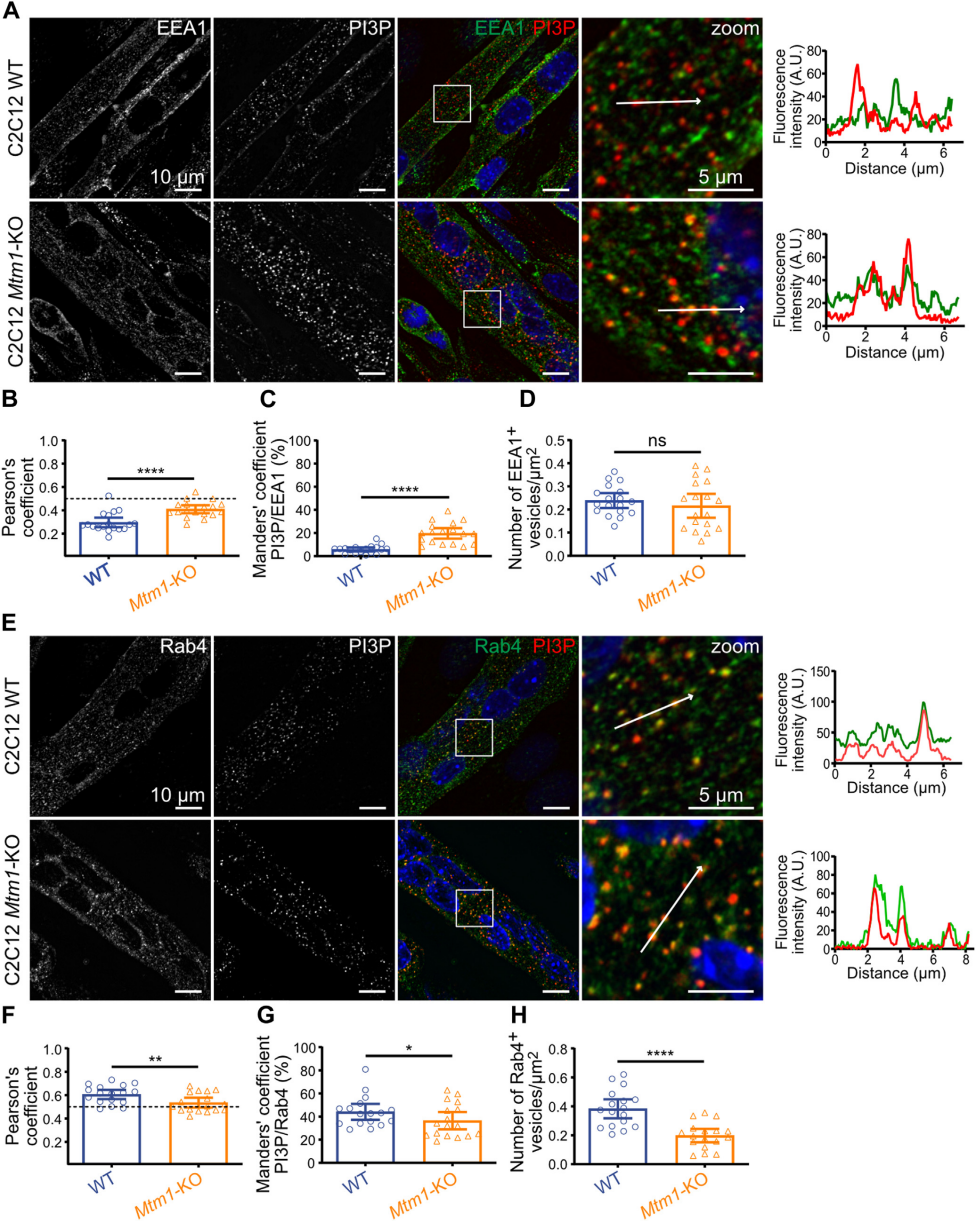
PI3P accumulates on EEA1-positive vesicles in the absence of MTM1 associated with defective Rab4-positive vesicle biogenesis. A: Representative confocal images of C2C12 WT and *Mtm1*-KO cells at 6 days of differentiation, labeled with a probe for PI3P (FYVE domain of Hrs protein), EEA1, and DAPI (blue). Scale bar represents 10 μm. Magnification of the boxed area shown on the right. Respective line scans are shown on the right of the zoom panel. Scale bar represents 5 μm. B: Quantification of Pearson's coefficient from the experiment described in A. Results are shown as mean ± 95% CI, n = 3, each point represents one field of view. ∗∗∗∗*P* < 0.0001 according to Student's *t*-test. C: Quantification of Manders' coefficient from the experiment described in A. Results are shown as mean ± 95% CI, n = 3, each point represents one field of view. ∗∗∗∗*P* < 0.0001 according to Student's *t*-test. D: Quantification of the number of EEA1-positive vesicles from the experiment described in A. Results are shown as mean ± 95% CI, n = 3, each point represents one field of view. ns = not significant according to Student's *t*-test. E: Representative confocal images of C2C12 WT and *Mtm1*-KO cells at 6 days of differentiation, labeled with a probe for PI3P (FYVE domain of Hrs protein), Rab4, and DAPI (blue). Scale bar represents 10 μm. Magnification of the boxed area shown on the right. Scale bar represents 5 μm. Respective line scans are shown on the right of the zoom panel. F: Quantification of Pearson's coefficient from the experiment described in E. Results are shown as mean ± 95% CI, n = 3, each point represents one field of view. ∗∗*P* < 0.01 according to Student's *t*-test. G: Quantification of Manders' coefficient from the experiment described in E. Results are shown as mean ± 95% CI, n = 3, each point represents one field of view. ns, not significant according to Student's *t*-test. H: Quantification of the number of Rab4-positive vesicles from the experiment described in E. Results are shown as mean ± 95% CI, n = 3, each point represents one field of view. ∗∗∗∗*P* < 0.0001 according to Student's *t*-test.

We subsequently colabeled PI3P along with EEA1 (Fig. 4A), a protein marker used for early endosome identification, which belongs to a group of elongated coiled-coil tethers possessing binding sites for both Rab5-GTP and PI3P. This property enables EEA1 to facilitate the connection between Rab5-GTP-positive vesicles and PI3P-containing endosomes, serving as the minimal component required for early endosome fusion (26). Analysis of the correlation between the two molecules using Pearson's coefficient indicated a weak colocalization similar to that observed with Rab5, but which increased in *Mtm1*-KO myotubes, with no overlap of the 95% CIs (Fig. 4B). Manders' overlap coefficient confirmed the accumulation of PI3P on EEA1-positive vesicles in *Mtm1*-KO myotubes, with no overlap of the 95% CI, suggesting that MTM1 hydrolyzes PI3P into PI on these vesicles (Fig. 4C). As for Rab5-positive vesicles, no difference in EEA1-positive vesicle density was observed in the absence of MTM1 (Fig. 4D).

Since neither of the two endosomal markers showed strong colocalization with PI3P, we proceeded to colabel PI3P with Rab4 (Fig. 4E), a protein associated with early endosomes and recycling vesicles. It plays a crucial role in recycling membrane components from early endosomes to the plasma membrane and operates at distinct sites from Rab5 within the early endosomal network (27). Pearson's coefficient indicated a strong colocalization between Rab4 and PI3P, implying that PI3P predominantly localizes on Rab4-positive vesicles (Fig. 4F). However, it does not seem to be the primary site of MTM1 action, as the absence of MTM1 does not increase the levels of PI3P on these vesicles (Fig. 4G). Nonetheless, a significant decrease in Rab4-positive vesicle density was observed, with no overlap of the 95% CI (Fig. 4H).

Overall, these results indicate that while PI3P is primarily found on Rab4-positive vesicles, MTM1 dephosphorylates PI3P on EEA1-positive vesicles, resulting in a disruption of Rab4-positive vesicle biogenesis.

### Knocking down *Pik3c2b* in *Mtm1*-KO cells counteracts the accumulation of PI3P on EEA1-positive vesicles

**Fig. 5:**
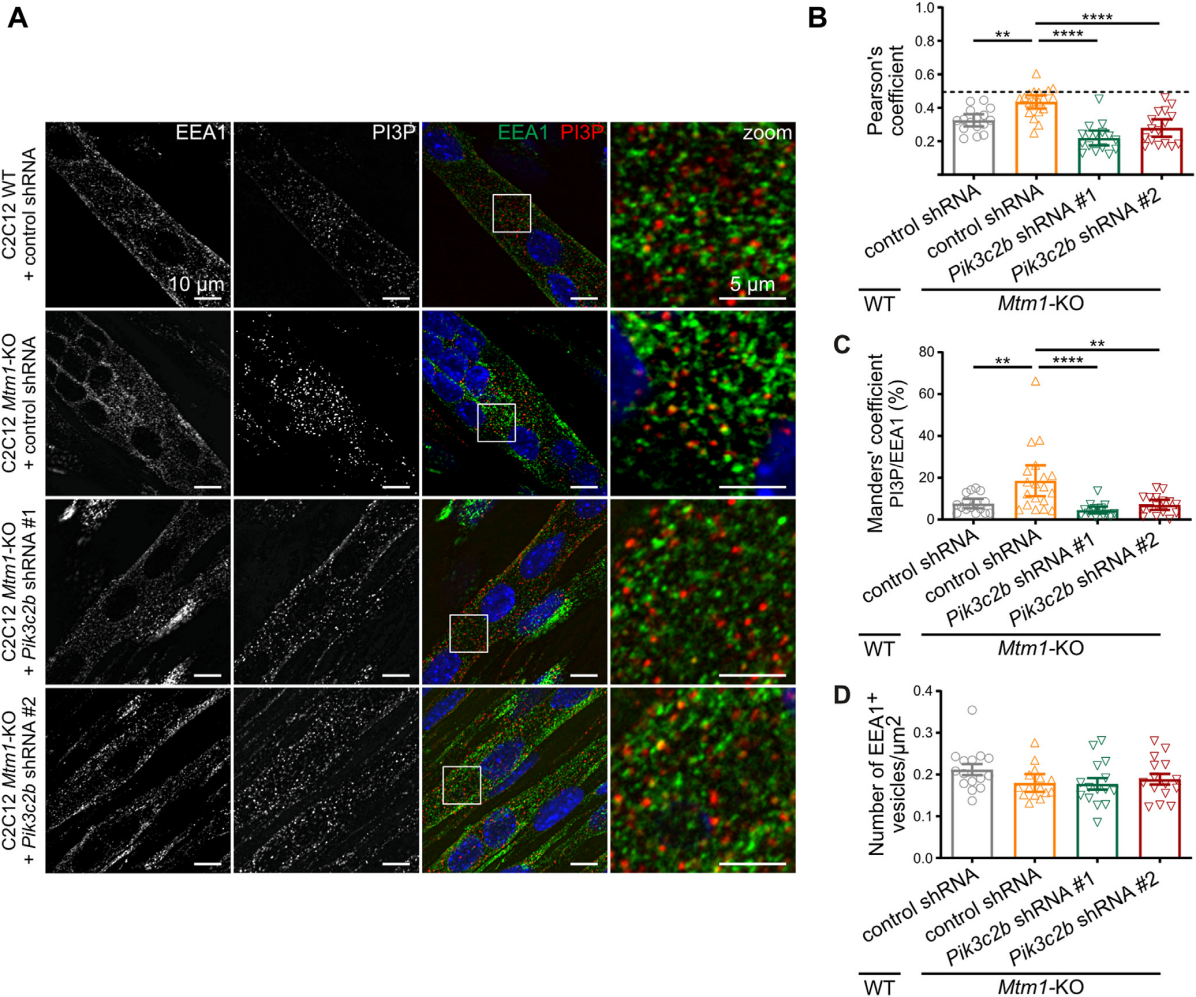
PI3KC2β extinction in *Mtm1*-KO cells counteracts the accumulation of PI3P on EEA1-positive vesicles. A: Representative confocal images of C2C12 WT and *Mtm1-*KO transduced control shRNA cells, and *Mtm1*-KO transduced with shRNAs targeting PI3KC2β, after 6 days of differentiation, labeled with a probe for PI3P (FYVE domain of Hrs protein), EEA1, and DAPI (blue). Scale bar represents 10 μm. Right panels show enlargements of the boxed areas. Scale bar represents 5 μm. B: Quantification of Pearson's coefficient from the experiment described in A. Results are shown as mean ± 95% CI, n = 3, each point represents one field of view. ∗∗*P* < 0.01 and ∗∗∗∗*P* < 0.0001 according to one-way ANOVA followed by Šídák's multiple comparison test. C: Quantification of the Manders' coefficient from the experiment described in A. Results are represented as mean ± 95% CI, n = 3, each point represents one field of view. ∗∗*P* < 0.01 and ∗∗∗∗*P* < 0.0001 according to one-way ANOVA followed by Šídák's multiple comparison test. D: Quantification of the number of EEA1-positive vesicles from the experiment described in A. Results are represented as mean ± 95% CI, n = 3, each point represents one field of view. Not significant according to one-way ANOVA followed by Šídák's multiple comparison test.

Since PI3P was found to accumulate on EEA1-positive vesicles in the absence of MTM1 (Fig. 4A–C), we tested whether the knockdown of *Pik3c2b* would decrease PI3P levels on these vesicles. We colabeled PI3P and EEA1 in WT and *Mtm1*-KO C2C12 myotubes expressing control shRNA, *Pik3c2b* shRNA #1, and *Pik3c2b* shRNA #2 (Fig. 5A). Analysis of Pearson's coefficient and Manders' overlap coefficients indicated that *Pik3c2b* depletion specifically reduced PI3P accumulation on EEA1-positive vesicles, restoring the percentage of PI3P on EEA1-positive vesicles to control levels observed in WT myotubes, with no overlap of the 95% CI (Fig. 5B, C). This reduction had no effect on the density of EEA1-positive vesicles (Fig. 5D). Therefore, these results indicate that MTM1 and PI3KC2β share the control of a common pool of PI3P on EEA1-positive vesicles, suggesting the involvement of specific binding partners for the coincidence detection of this PI3P pool. To further support our conclusions regarding the roles of MTM1 and PI3KC2β in regulating the EEA1-associated PI3P pool, we overexpressed HA-PI3KC2β or FLAG-MTM1 in HeLa cells (supplemental Figs. S1 and S3A). In these cells, PI3P was highly enriched on EEA1-positive vesicles. Overexpression of HA-PI3KC2β significantly increased the colocalization between EEA1 and PI3P, whereas even low levels of FLAG-MTM1 overexpression led to a marked reduction. Notably, in highly transfected cells, PI3P signals were undetectable (supplemental Fig. S3B–D). These results support that PI3KC2β promotes PI3P accumulation on EEA1-positive vesicles, whereas MTM1 counteracts this effect by dephosphorylating PI3P.

### Extinction of *Pi3kc2b* in *Mtm1*-KO myotubes restores Rab4-positive vesicle numbers

**Fig. 6:**
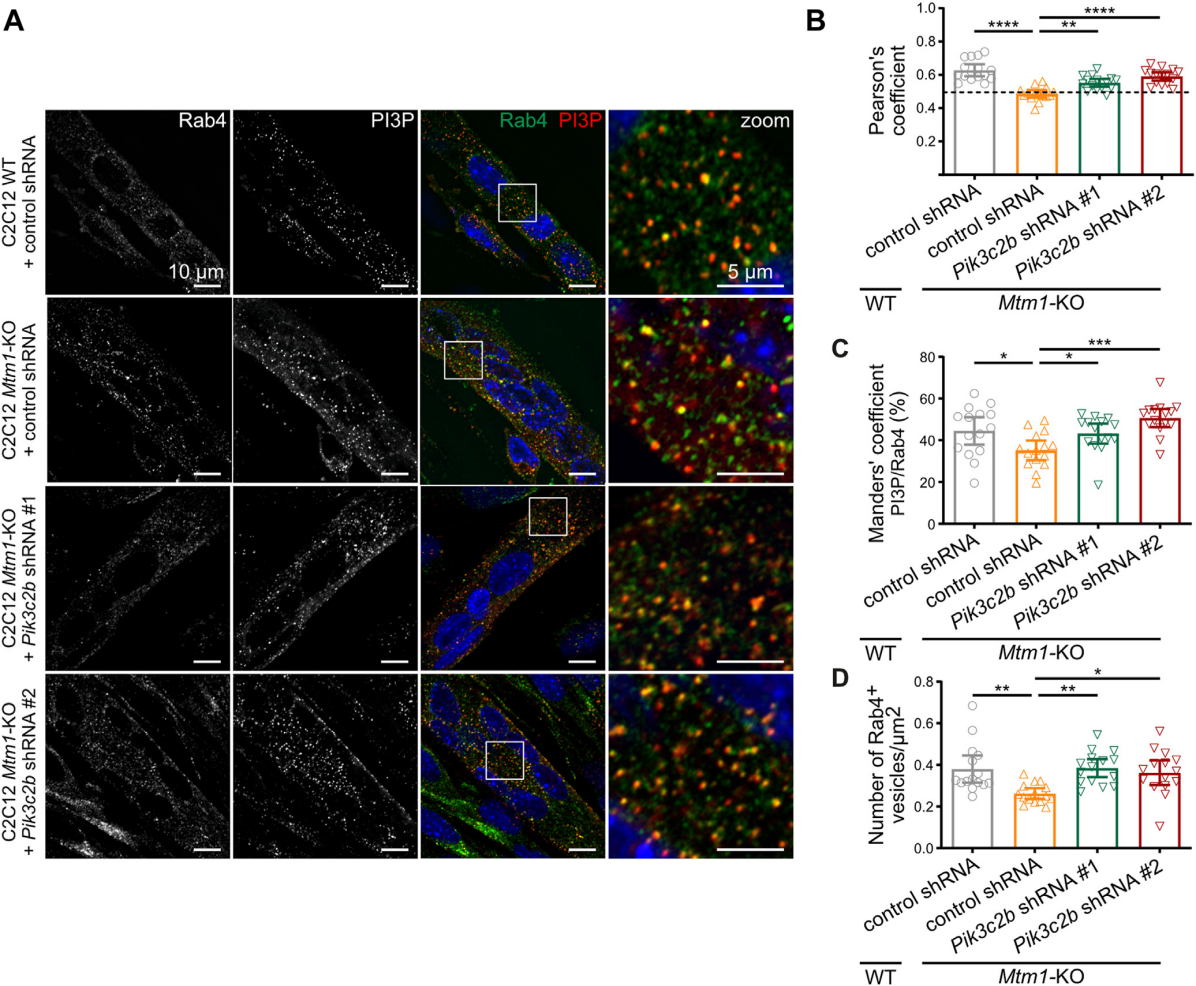
PI3KC2β extinction in *Mtm1*-KO cells restores Rab4-positive vesicle numbers. A: Representative confocal images of C2C12 WT and *Mtm1-*KO transduced control shRNA cells, and *Mtm1*-KO transduced with shRNAs targeting PI3KC2β, after 6 days of differentiation, labeled with a probe for PI3P (FYVE domain of Hrs protein), Rab4, and DAPI (blue). Scale bar represents 10 μm. Right panels show enlargements of the boxed areas. Scale bar represents 5 μm. B: Quantification of Pearson's coefficient from the experiment described in A. Results are shown as mean ± 95% CI, n = 3, each point represents one field of view. ∗∗*P* < 0.01 and ∗∗∗∗*P* < 0.0001 according to one-way ANOVA followed by Šídák's multiple comparison test. C: Quantification of the Manders' coefficient from the experiment described in A. Results are represented as mean ± 95% CI, n = 3, each point represents one field of view. ∗*P* < 0.05 and ∗∗∗*P* < 0.001 according to one-way ANOVA followed by Šídák's multiple comparison test. D: Quantification of the number of Rab4-positive vesicles from the experiment described in A. Results are represented as mean ± 95% CI, n = 3, each point represents one field of view. ∗*P* < 0.05 and ∗∗*P* < 0.01 according to one-way ANOVA followed by Šídák's multiple comparison test.

We then examined the impact of *Pik3c2b* extinction in *Mtm1*-KO cells on the Rab4-positive compartment, in which we observed a slight decrease in PI3P levels (Fig. 4E–G), accompanied by a reduction in vesicle density (Fig. 4H). Colabeling analysis of Rab4 and PI3P in WT and *Mtm1*-KO C2C12 myotubes expressing control shRNA, *Pik3c2b* shRNA #1, and *Pik3c2b* shRNA #2 (Fig. 6A) confirmed that, although MTM1 does not dephosphorylate PI3P on Rab4-positive vesicles, these vesicles exhibit lower levels of PI3P. *Pik3c2b* extinction restored PI3P levels on these vesicles (with overlap of the 95% CI for Manders' coefficients) (Fig. 6B, C), suggesting that the accumulation of PI3P on the EEA1-positive compartment modifies the homeostasis of PI3P on the Rab4 compartment. Interestingly, *Pik3c2b* depletion restored the number of Rab4-positive vesicles in *Mtm1*-KO myotubes (with no overlap of the 95% CI), indicating that MTM1, together with PI3KC2β, tightly regulates the biogenesis of these vesicles (Fig. 6D).

## Discussion

Our study confirms the critical role of MTM1 in phosphoinositide metabolism, specifically its impact on the regulation of PI3P within endosomal compartments. Using an *Mtm1*-KO skeletal muscle cell line that recapitulates the pathological features of XLMTM, we provided new insights into the cellular mechanisms underlying this disease. Contrary to other cell models where PI3P is primarily localized on Rab5-positive vesicles and is less abundant in the Rab4 subcompartment (24), our results indicate that in myotubes, PI3P predominantly localizes on Rab4-positive vesicles. This suggests that skeletal muscle cells may use a different PI3P-dependent pathway for vesicle trafficking, potentially reflecting unique functional requirements in muscle physiology.

However, the absence of MTM1 leads to an accumulation of PI3P on EEA1-positive endosomes, indicating that MTM1 is essential for the proper turnover of this phosphoinositide in early endosomes. Interestingly, our results underscore the importance of Rab4-positive vesicles in the pathology of XLMTM. Indeed, in *Mtm1*-KO myotubes, there is a significant impairment in the biogenesis of Rab4-positive vesicles, evidenced by a substantial reduction in their density. This finding suggests a novel role for MTM1 in regulating the recycling of endosomal cargo *via* Rab4-positive vesicles. Notably, this difference was not observed in myoblasts (supplemental Fig. S4), where MTM1 is expressed at low levels (18, 28), highlighting the importance of MTM1 in this phenotype. However, we observed that PI3P was less enriched on EEA1-positive vesicles in *Mtm1*-KO myoblast (supplemental Fig. S4B).

Importantly, the rescue of *Mtm1*-KO phenotypes by PI3KC2β depletion confirms the therapeutic potential of targeting PI3P metabolism in XLMTM (12, 17). Our data demonstrate that PI3KC2β depletion not only normalizes PI3P levels on EEA1-positive endosomes but also restores the density of Rab4-positive vesicles, thereby correcting the aberrant endosomal trafficking observed in *Mtm1*-KO myotubes.

In conclusion, these results highlight the interdependent roles of MTM1 and PI3KC2β in maintaining endosomal homeostasis in skeletal muscle cells. Our findings provide a deeper understanding of the molecular mechanisms disrupted in XLMTM. We establish that MTM1 is pivotal for the regulation of PI3P within early endosomes and that its loss disrupts the normal biogenesis of Rab4-positive vesicles, possibly impairing cargo recycling. The successful rescue of *Mtm1*-KO phenotypes by PI3KC2β depletion further show that this cell line could be used for inhibitor screening and opens new avenues for therapeutic intervention in XLMTM. Future studies should elucidate the molecular interplay between MTM1 and PI3KC2β.

## Data availability

All data are available in the main text or the supplemental data.

## Supplemental data

This article contains supplemental data.

## Conflict of interest

The authors declare that they have no conflicts of interest with the contents of this article.
