# Supplementary material for: PI3KC2β depletion rescues endosomal trafficking defects in Mtm1 knockout skeletal muscle cells
Source: J Lipid Res. 2025 Feb 12;66(3):100756. doi: 10.1016/j.jlr.2025.100756 (PMC11930147; doi:10.1016/j.jlr.2025.100756)
Supplement: Supplementary data [file mmc1.pdf]

Supplementary data

PI3KC2β depletion rescues endosomal trafficking defects in *Mtm1* knockout skeletal muscle cells

Mélanie Mansat [1, †], Afi Oportune Kpotor [1, †], Anne Mazars [1], Gaëtan Chicanne [1], Bernard Payrastre [1,2] and Julien Viaud [2, *]

[1] INSERM UMR1297, University of Toulouse 3, Institute of Metabolic and Cardiovascular Diseases (I2MC), Avenue Jean Poulhès, BP 84225, 31432 Toulouse Cedex 04, France.

[2] University Hospital of Toulouse, Hematology Laboratory, 31059 Toulouse Cedex 03, France.

[†] These authors contributed equally to this work.

***** For correspondence: Julien Viaud, INSERM UMR1297, University of Toulouse 3, Institute of Metabolic and Cardiovascular Diseases (I2MC), Avenue Jean Poulhès, BP 84225, 31432 Toulouse Cedex 04, France. Tel: +33 531224151, E-mail: julien.viaud@inserm.fr

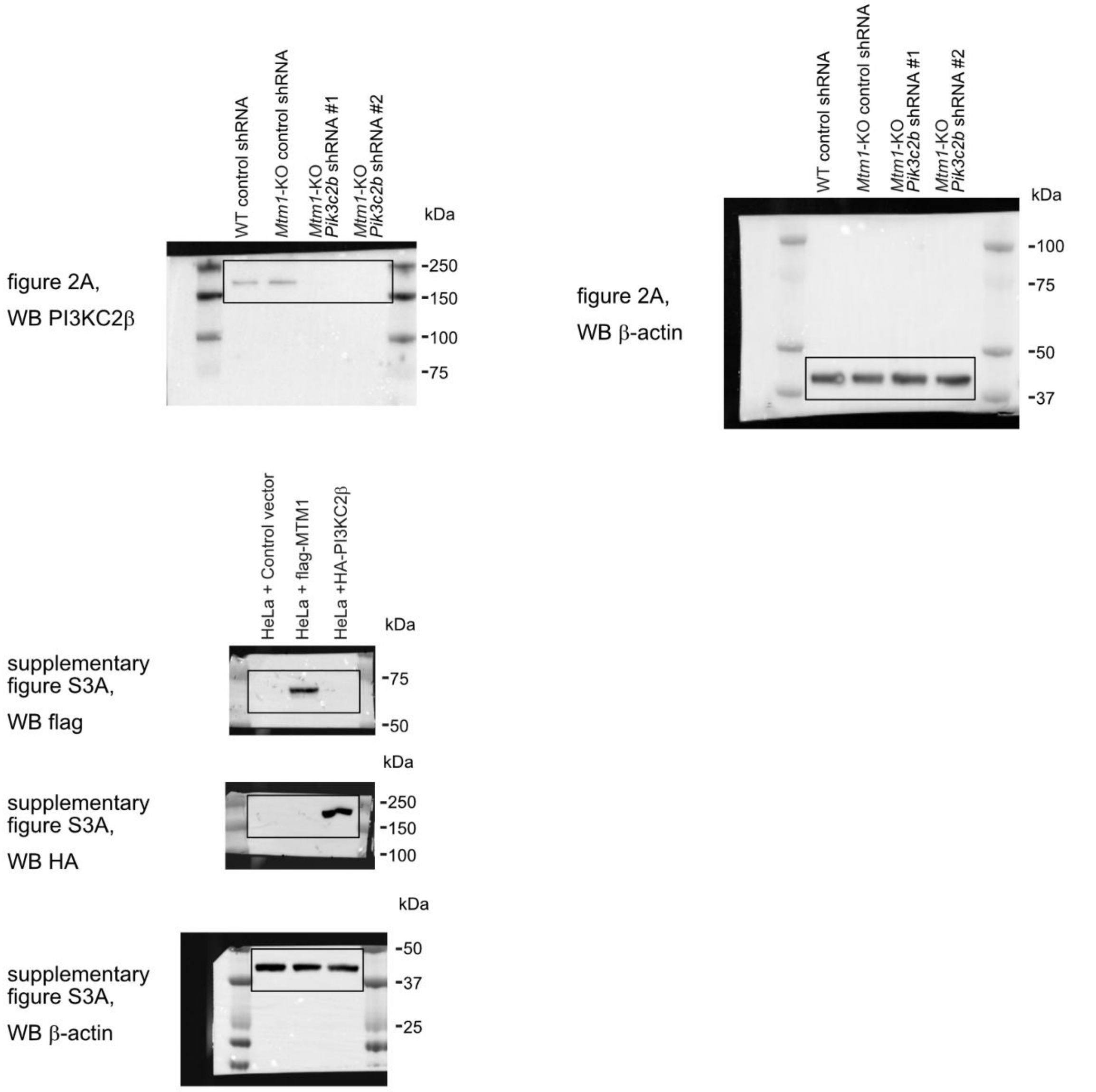

Fig. S1. Uncropped western blot images from figure 2 and supplementary Fig. S3.

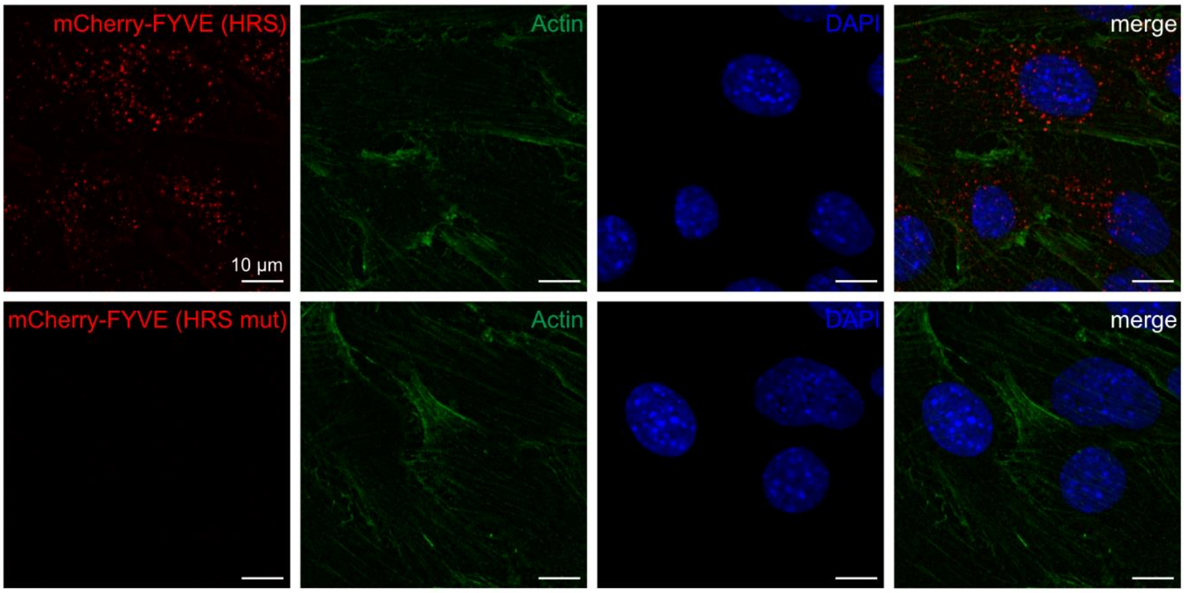

Fig. S2. Top: Representative images of PI3P labeling with the mCherry-FYVE (HRS) probe in C2C12 WT myoblast. Bottom: Representative images of PI3P labeling with the mCherry-FYVE (HRS mut) probe in C2C12 WT myoblast. The mutations consist of R24A/K25A/R29A. Scale bar, 10 μm.

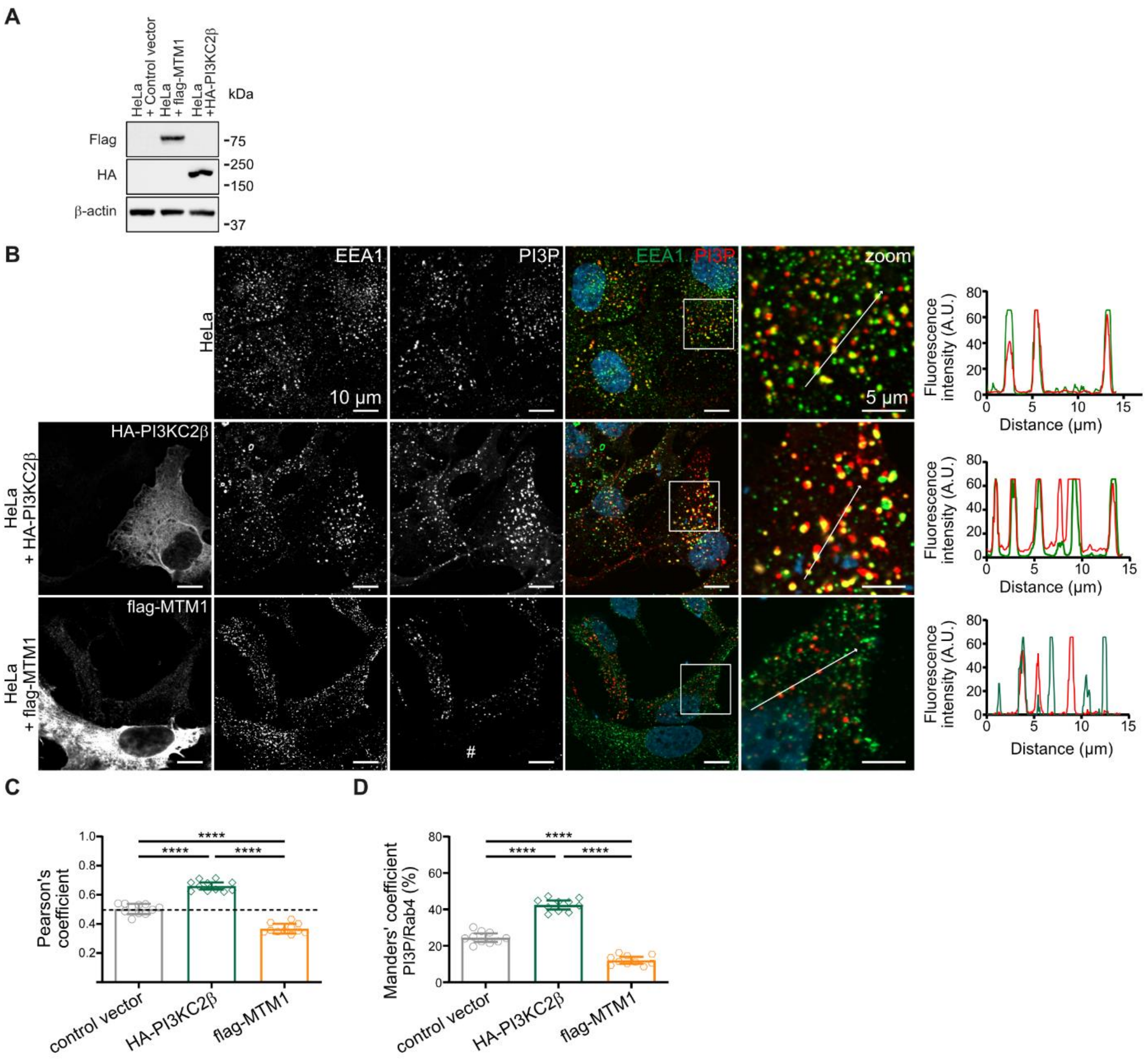

Fig. S3. PI3P accumulates on EEA1-positive vesicles in HeLa cells expressing HA-PI3KC2β, while flag-MTM1 overexpression leads to a marked reduction. A: Expression of HA-PI3KC2β and flag-MTM1 in HeLa cells transfected with plasmids encoding these constructs, analyzed by Western blot. Images are representative of three independent experiments. B: Representative confocal images of Hela cells expressing or not expressing HA-PI3KC2β or flag-MTM1, labeled with a probe for PI3P (FYVE domain of Hrs protein), EEA1, and DAPI (blue). Scale bar: 10 µm. # indicates a cell highly expressing flag-MTM1, in which PI3P is not detected. Magnification of the boxed area shown on the right. Respective line scans are shown on the right of the zoom panel. Scale bar: 5 µm. C: Quantification of Pearson's coefficient from the experiment described in B. For flag-MTM1-expressing cells, only those with low expression levels were considered. Results are shown as mean ± 95% confidence interval, n=3, each point represents one field of view. ****$p<0.0001$ according to one-way ANOVA followed by Šídák's multiple comparison test. D: Quantification of the Manders' coefficient from the experiment described in B. Results are represented as mean ± 95% confidence interval, n=3, each point represents one field of view. ****$p<0.0001$ according to one-way ANOVA followed by Šídák's multiple comparison test.

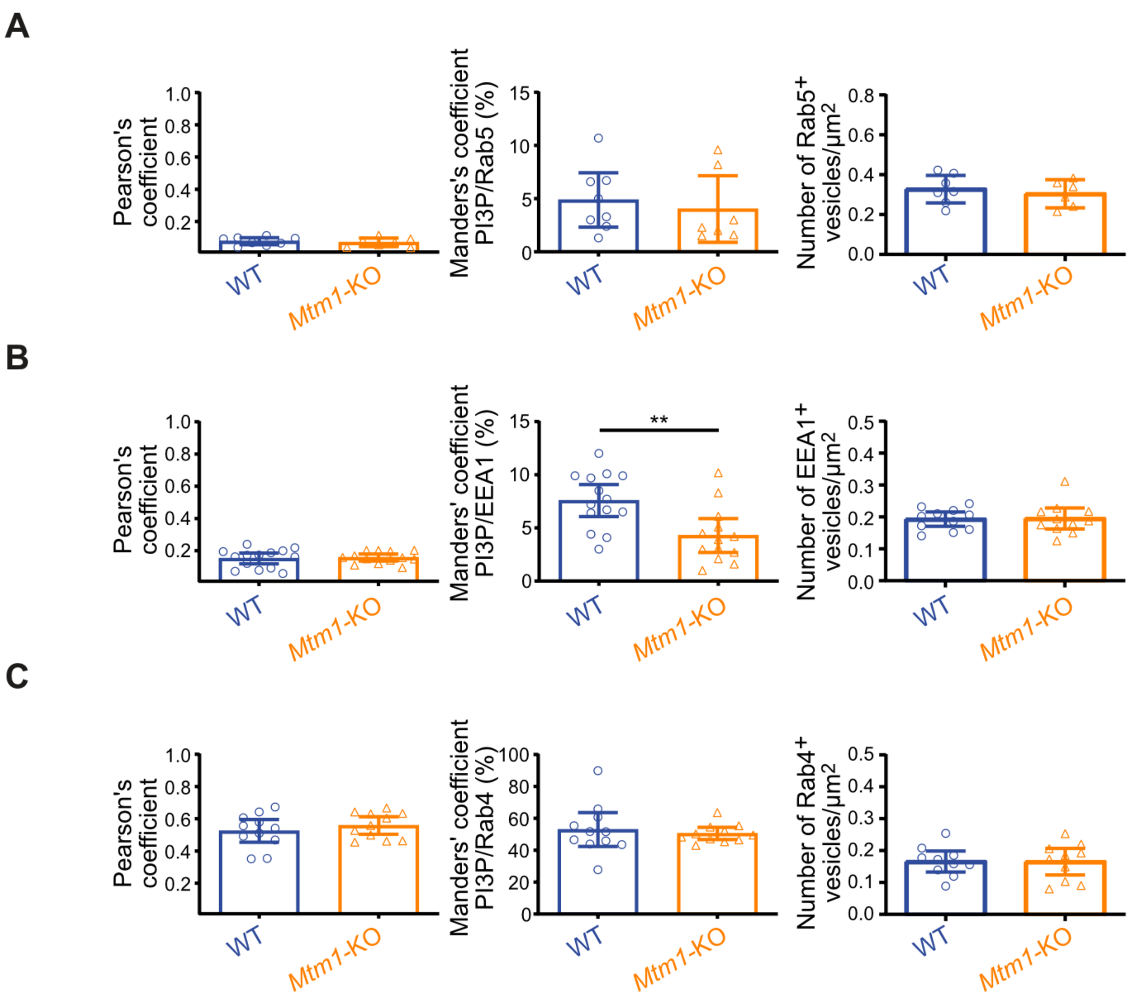

Fig. S4. Quantification of Pearson's coefficient, Manders' coefficient, and the number of indicated positive vesicles in WT and *Mtm1*-KO myoblasts labeled with a probe for PI3P (FYVE domain of Hrs protein) and the indicated endosomal marker. A: Rab5. B: EEA1. C: Rab4. Results are shown as mean ± 95% confidence interval, n=3, each point represents one field of view. $**p<0.01$ according to Student's t-test.
